# Changing self-concept in the time of COVID-19: a close look at physician reflections on social media

**DOI:** 10.1186/s13010-021-00113-x

**Published:** 2022-01-26

**Authors:** Min Chiam, Chong Yao Ho, Elaine Quah, Keith Zi Yuan Chua, Caleb Wei Hao Ng, Elijah Gin Lim, Javier Rui Ming Tan, Ruth Si Man Wong, Yun Ting Ong, Yoke Lim Soong, Jin Wei Kwek, Wei Sean Yong, Kiley Wei Jen Loh, Crystal Lim, Stephen Mason, Lalit Kumar Radha Krishna

**Affiliations:** 1 Division of Cancer Education, National Cancer Centre Singapore, 11 Hospital Crescent, Singapore, 169610 Singapore; 2 Yong Loo Lin School of Medicine, National University of Singapore, NUHS Tower Block, 1E Kent Ridge Road, Level 11, Singapore, 119228 Singapore; 3 Division of Supportive and Palliative Care, National Cancer Centre Singapore, 11 Hospital Dr, Singapore, 169610 Singapore; 4 Division of Radiation Oncology, National Cancer Centre Singapore, 11 Hospital Crescent, Singapore, 169610 Singapore; 5 Division of Oncologic Imaging, National Cancer Centre Singapore, 11 Hospital Crescent, Singapore, 169610 Singapore; 6 Division of Surgical Oncology, National Cancer Centre Singapore, 11 Hospital Crescent, Singapore, 169610 Singapore; 7 Medical Social Services, Singapore General Hospital, Outram Rd, Singapore, 169608 Singapore; 8 Palliative Care Institute Liverpool, Academic Palliative & End of Life Care Centre, University of Liverpool, Liverpool, UK; 9 Cancer Research Centre, University of Liverpool, 200 London Rd, Liverpool, L3 9TA UK; 10 Duke-NUS Medical School, National University of Singapore, 8 College Rd, Singapore, 169857 Singapore; 11 Centre of Biomedical Ethics, National University of Singapore, 21 Lower Kent Ridge Rd, Singapore, 119077 Singapore; 12 PalC, The Palliative Care Centre for Excellence in Research and Education, PalC c/o Dover Park Hospice, 10 Jalan Tan Tock Seng, Singapore, 308436 Singapore

**Keywords:** COVID-19, Physicians, Reflections, Personal experiences, Social media, Self-concept, Personhood, Ring Theory of Personhood

## Abstract

**Background:**

The COVID-19 pandemic has changed the healthcare landscape drastically. Stricken by sharp surges in morbidity and mortality with resource and manpower shortages confounding their efforts, the medical community has witnessed high rates of burnout and post-traumatic stress amongst themselves. Whilst the prevailing literature has offered glimpses into their professional war, no review thus far has collated the deeply personal reflections of physicians and ascertained how their self-concept, self-esteem and perceived self-worth has altered during this crisis. Without adequate intervention, this may have profound effects on their mental and physical health, personal relationships and professional efficacy.

**Methods:**

With mentions of the coronavirus pervading social media by the millions, this paper set out to collate and thematically analyse social media posts containing first-person physician reflections on how COVID-19 affected their lives and their coping mechanisms. A consistent search strategy was employed and a PRISMA flowchart was used to map out the inclusion/exclusion criteria.

**Results:**

A total of 590 social media posts were screened, 511 evaluated, and 108 included for analysis. Salient themes identified include Disruptions to Personal Psycho-Emotional State, Disruptions to Professional Care Delivery, Concern for Family, Response from Institution, Response from Society and Coping Mechanisms.

**Conclusion:**

It is evident that the distress experienced by physicians during this time has been manifold, multi-faceted and dominantly negative. Self-concepts were distorted with weakened self-esteem and perceived self-worth observed. The Ring Theory of Personhood (RToP) was adopted to explain COVID-19’s impact on physician personhood as it considers existential, individual, relational and social concepts of the self. These entwined self-concepts serve as ‘compensatory’ to one another, with coping mechanisms buffering and fortifying the physician’s overall personhood. With healthcare institutions playing a vital role in providing timely and targeted support, it was further proposed that a comprehensive assessment tool based on the RToP could be developed to detect at-risk physicians and evaluate the presence and effectiveness of established support structures.

**Supplementary Information:**

The online version contains supplementary material available at 10.1186/s13010-021-00113-x.

## Introduction

The COVID-19 global pandemic has raged devastations on economies, social practices and have overwhelmed healthcare systems like never before in modern times [1–4]. Unprecedented waves of morbidity and mortality have seen a whirlwind of attempts to contain the infection amidst shortages in manpower and personal protective equipment [5, 6]. With their professional responsibilities pushed to the fore, it is perhaps easy to take for granted the profound humanity of these healthcare workers and the acute distress they are called to shoulder. Rates of burnout and post-traumatic stress have been astronomical [7–10]. Although * BBC* interviews [11, 12] and various podcasts [13, 14] have provided glimpses into their harrowing professional lives, no review conducted thus far has given sufficient voice to their deeply personal stories - in particular, how the crisis has affected their perceptions of themselves. This notion of self-concept is significant as it is tied closely to self-image, self-esteem, and perceived self-worth [15–19]. Without adequate intervention, this may have longitudinal ramifications on their mental and physical health, personal relationships and professional efficacy [20–23].

As George Agich observed, reflections serve as the intellectual concomitant of adaptive and coping behaviours in the face of internal and external disruptions to habitual routines [24]. On 28 February 2020 alone, *Forbes *reported that 6.7 million people mentioned coronavirus on social media [25] and analytics company *Sprinklr* reported over 19 million mentions of coronavirus-related terms just weeks later [26]. Some of these were from personal reflections written by physicians. This paper thus set out to collate social media posts containing first-person physician reflections on how the precarious climate has affected their personal and professional lives. In tandem, it looked at the coping mechanisms that have fortified them. In the process, the paper identified a key conceptual framework that may elucidate ways in which healthcare institutions could better proffer timely and targeted support.

## Methodology

### Searching

The research team searched for the keywords, ‘physicians’, ‘COVID-19’, ‘experience’, ‘reflection’, ‘lives’ and their permutations and focused on the following social media platforms: YouTube, Twitter, Instagram, Facebook, Reddit, KevinMD and Medium. Physicians were limited to those involved in the care of COVID-19 and/or non-COVID-19 patients both in-person and/or through telemedicine during the pandemic. Only first-person reflections contained within social media posts including those from personal interviews and published between 1 January 2020 and 26 May 2020 were selected in line with increasing global awareness of the virus. To accommodate time constraints faced by the research team, only social media posts in the form of written text or audio-visual video, and in English or translated into English, were included. No restrictions were placed on geographical location and searches were carried out between 21 May 2020 and 26 May 2020. The search strategy may be found in Additional file 1: Appendix A.

### Extracting and charting results

Sandelowski and Barroso's (2006) [27] approach to ‘negotiated consensual validation’ was used to achieve consensus on the final list of social media posts through reiterative and collaborative discussions between team members. A total of 590 posts were screened, 511 full posts evaluated, and 108 full posts included for analysis. The PRISMA flowchart which maps out the inclusion/exclusion criteria may be found in Additional file 1: Appendix B.

Whilst dominantly from the United States, social media posts from physicians based in the United Kingdom, Australia, Philippines, Singapore, China and India were also included. The data extracted and charted were garnered from physicians from Emergency Medicine, Critical Care, Anaesthesiology, Internal Medicine, Family Medicine, Obstetrics and Gynaecology, Paediatrics and Psychiatry.

### Ethics

This study was granted exemption by the SingHealth Centralised Institutional Review Board (CIRB Ref 2020/3055: Changing Self-concept in the Time of COVID-19: A Close Look at Physician Reflections on Social Media). Whilst data was gathered from publicly available domains, concerted efforts were made to remove personal identifiers through searches on Google and the social media platform that the data was derived from. Identifiable direct quotations were presented only where cited in news articles or interviews.

## Results

### Physicians’ experiences

Salient themes from these reflections have been grouped into the following: Personal Psycho-Emotional State, Professional Care Delivery, Concern for Family, Response from Institution, Response from Society and Coping Mechanisms.

### Disruptions to personal psycho-emotional state

#### Confrontation with own susceptibility and mortality

With hospital wards saturated with the critically ill and dying, physicians observed a heightened confrontation with their own mortality, their patients’ cadaverous bodies presaging their own human fallibility. ‘You walk in and you can smell fear, you can smell death,’ one described. Whilst many likened the raging COVID-19 devastations to a war zone, persistent risk of their own infection during patient care delivery saw them ruefully reflect that they did not enter medicine to risk their lives. With one resigning himself to the fact that it was only a matter of time before he fell victim to the disease, others formulated their contingency plans. ‘We’ve talked about who gets our pets…which is somewhat of an easier discussion than who gets your children,’ one bitterly noted.

#### Pervasive guilt

Guilt was also commonplace amongst healthcare workers. In particular, those who were not shoulder to shoulder on the frontlines agonised over their own ineffectuality, guilt-ridden for voicing their hardships in their better off positions. For one ER physician, feelings of remorse persisted even though her late term pregnancy necessitated her stepping away from her overwhelmed team. One observed the double bind of the profession – that absence from family and dying patients alike evoked feelings of culpability.

#### Overwhelming sorrow

Amidst mounting uncertainties, staggering caseloads and having to watch patients die alone, permutations of the word ‘heartbreaking’ surfaced repeatedly. Some physicians expressed their grief through moments of tears with one ER physician crying for the ones who had died, for the families who could not see them die and for the ones waiting in the lobby yet to know of their demise. Many described their sorrow and weeping as unprecedented with some left deeply distraught and unable to contain their emotions in front of their patients.

#### Sacrificing self-care

Finding avenues for stress relief proved difficult as incessant exposure to patients on the verge of crashing led to prolonged periods on-call, with some shifting into autopilot and experiencing a sense of depersonalisation. With all aspects of life, social media and medicine focused on the COVID-19 pandemic, one physician acknowledged that her struggles were rooted in her inability to disconnect due to the pandemic's intrusive presence. Lack of time for self-care for another saw rashes burgeoning all over his chest, neck and arms. Whilst this lack of reprieve was recognised as unsustainable, one acknowledged that her sleep deprivation and lack of time off were products of her own choosing, a necessary trade-off.

### Disruptions to professional care delivery

#### Sense of unpreparedness, helplessness and inadequacy

This tumultuous climate also saw helplessness pervade the wards with physicians grappling with their own appraisals of their professional adequacy. One physician described his inability to stop COVID-19’s devastating disease progression in his patients as akin to watching a train crash played out in slow motion. Even critical care specialists armoured with the experience of witnessing the sickest of the sick in their day to day were left feeling ‘out of their wheelhouse’, untrained and unprepared. With contradicting recommendations leaving them disoriented, piecemeal knowledge on the virus led to poignant statements on their feelings of ‘impotence’ and ‘futility’ as well as questions as to whether they had made a difference at all.

#### Adaptations and disruptions

The speed at which adaptations to clinical practice was required of physicians proved to be physically and psychologically demanding. With the persistent need to don their personal protective equipment, some experienced dehydration, headaches, painful abrasions and feelings of claustrophobia. A move towards telemedicine also led to back-to-back schedules which heavily fatigued some. One psychiatrist who conducted consultations from home saw the blurring of her home and work life. Yet, multiple physicians notably reported greater disconnect from their patients, either due to the lack of human touch afforded by these online interactions or their diminished personability when suited up in protective gear. One described a dementia patient cowering in fear and wailing upon seeing her – ‘I wasn’t human,’ she observed. The distortion of their faces, muffling of their voices and inability to maintain eye contact intervened with their duty and desire to care and comfort. Fear for their own lives also saw physicians taking pause – ‘it is harder to save your patients’ lives when you are trying to save your own,’ one explained. Conversely, some deeply worried that they would infect and compromise the safety of the vulnerable.

#### Fraught ethical decision-making

Most pertinently, scarcity in manpower, ventilators, beds in the intensive care unit and key medications such as sedatives and opioids saw the need for wartime triage and fraught ethical decision-making. The question as to whether to abide by the rhetoric of first come first served or ‘apply a cruel and horrible utilitarian calculus’ in the discerning of who to treat or let die saw some physicians reiterating their moral distress – ‘I definitely did not go into the practice of medicine to play God and nor do I want to’. In addition, with more healthcare workers required to be in the thick of things, team leaders were faced with the difficult dilemma of sending their colleagues into close contact with COVID-19 patients without sufficient protection. A heavy weight that led to feelings of both terror and guilt, one senior physician voiced her desire to make contact with COVID-19 cases volunteer-only, that she would otherwise see to these patients herself in order to protect her people.

### Concern for family

#### Worry for close relations

With the tenacious spread of COVID-19, many physicians expressed deep worry for their close relations – close colleagues, friends and family. Whilst some were terrified at the eventuality of receiving news that their good friends on the frontlines had died, for others, the lonely deaths of their patients culminated in a transference of fear. One new mother reportedly suffered from ‘crippling anxiety’ whenever anyone came into close proximity to her immunocompromised newborn child. Another recalled deliriously pleading with her husband not to die. Due to the high-risk nature of their job, some worried about contagion risk to older family members who were more susceptible to infection, morbidity and mortality. Some physicians resorted to stripping off their scrubs and changing in the hospital parking lot before returning home.

#### Enforced self-isolation

To reduce compromising their loved ones, many placed themselves under enforced isolation, ‘trading one touch for another’ as patients became their primary source of human contact. Whilst one psychiatrist made the difficult decision to stop breastfeeding, another critical care physician voluntarily left home to safeguard his family – sleeping in his car, the hospital call room, and finally a tent in his garage, uncertain as to how long it would be his homestay. Such self-quarantine measures also led to important family milestones being missed with one internist lamenting having only caught his son’s first steps through an iPad – ‘I desperately...wanted to be the person he was walking toward’.

#### Disruptions to family arrangements and rituals

With mass closure of stores and services, one single mother bemoaned that her inability to find childcare arrangements interfered with her professional commitments. Social distancing measures and travel restrictions also saw funeral rituals, meant to be a ‘ceremony of a celebration of life’, severely modified, shortened or carried out in their absence. One mourned the knowledge that her mother, diagnosed with COVID-19, had died at an assisted living facility out of state, her burial attended by just ten people, without her.

### Response from institution

#### Failure in duty to protect

Expected to toil in hazardous settings for long hours without adequate protective equipment, many believed that the duty of care they were owed was severely lacking under the current for-profit medical system. Wondering if their Hippocratic oaths equated to a moral duty that would not see them treated in kind, one perceived himself as a commodity, an expendable cog within the machinery of the larger economy. Instructed to wear trash bags and bandanas in place of proper medical gear, many physicians felt undervalued, ill-protected and let down. Likening the pandemic to an incoming tsunami, one physician expressed dismay that a life buoy was all that their federal government had offered. The prospect of being detained for whistleblowing also underscored the skewed priorities of government bodies in placing national image over safety.

### Response from society

#### Ignorance and flippancy

Due to a pervasive fear of the hospital environment, late presentations of non-COVID-19 conditions in patients resulted in a sharp increase in complex emergency cases, further overwhelming physicians. The questionable veracity of travel declarations made by patients also resulted in much distress as these implicated safety and triaging procedures. The casual ignorance and lack of adherence to social distancing measures by members of the wider society also caused physicians much grief as they threatened to nullify progress made with containing the spread of infection. In turn, the spread of misinformation, labelling of COVID-19 as a politically-driven hoax and aggressive protests for freedom not only undermined their concerted healthcare efforts but trivialised their pain and the innocent deaths they were forced to bear witness to.

#### Overt hostility and racism

Some healthcare workers were reportedly viewed as a health threat and thus shunned by the public, verbally and physically abused with their children told not to return to school. The wave of anti-Chinese sentiment that reverberated across the globe also saw a plethora of ethnically Asian physicians reflecting upon the rampant racism they experienced and witnessed in their community. These ranged from microaggressions to threatened or explicit harm. Slanderous accusations of being ‘a disgusting, filthy bat-eater’ and ‘a selfish disease carrier’ saw further disparaging remarks that they had no right to be in their country. One physician described his ordeal as having made him hyperaware of himself – ‘the racism conjured by COVID-19 has made it impossible to forget my Asian self when with my patients. I am now highly conscious of who I am’.

### Coping mechanisms

In response, physicians described various coping strategies that they either personally adopted or recognised as useful in alleviating their anguish (Table 1). Communal support offered by friends and family, fellow healthcare professionals and the wider society were also seen from these reflections as having bolstered them in their time of duress (Table 2).

**Table 1:** Personal coping strategies discerned from physicians’ social media reflections

Personal Coping Strategies	
**Taking Action**	
**Speaking out** ▪ Expressing personal emotions and grievances ▪ Honouring and mourning deaths of colleagues and patients ▪ Emphasising shared humanity and taking a stand against discrimination ▪ Spreading awareness of COVID-19’s severity ▪ Sharing COVID-19 information and dispelling myths ▪ Illuminating ways in which healthcare workers could be directly supported	
**Practising self-care by disconnecting from medicine** ▪ Distancing from social media ▪ Engaging in religious activities and prayer ▪ Immersing in hobbies such as exercise, reading and other home projects ▪ Indulging in humour by creating light-hearted videos and viral challenges	
**Adapting with an open mind** ▪ Using e-platforms to broach physical distance with loved ones ▪ Developing novel ways to comfort and communicate with patients ▪ Upskilling and learning to refashion medical equipment ▪ Supporting intensive care units as a volunteer ▪ Serving as an informal mentor to junior members of the healthcare team	
**Reframing Thoughts**	
**Validating one’s own:** ▪ Actions as morally necessary ▪ Personal strengths such as resourcefulness and resilience ▪ Past experiences as a source of knowledge and preparedness ▪ Professional commitment to caring for patients ▪ Professional duty as offering life purpose and satisfaction	
**Focusing on gratitude for:** ▪ Accessibility to personal protective equipment ▪ Job security ▪ Family’s health and safety ▪ Opportunities to witness patients recover ▪ Opportunities to work in a dynamic and skillful team ▪ Colleagues working on the frontlines

**Table 2:** Communal support discerned from physicians’ social media reflections

Communal Support	
**Family and friends** ▪ Offering emotional outlets and confidential avenues for sharing of experiences	
**Fellow healthcare professionals** ▪ Providing intercollegial solidarity ▪ Role modelling through acts of selflessness and fearlessness ▪ Reigniting and encouraging hope ▪ Infusing work environment with humour and positivity ▪ Daily check-ins by wellness teams drawing attention to importance of mental health	
**Wider society** ▪ Online support groups offering advice and validating personal anxieties ▪ Essential workers in the service industry helping to keep public spaces safe ▪ Community members showing appreciation for healthcare efforts ▪ Community members donating food and medical resources	

## Discussion

### All-consuming effects on personhood

From these reflections, it is clear that the distress experienced by physicians during the COVID-19 pandemic has been manifold, multi-faceted and dominantly negative. Indeed they resonate with findings from prevailing literature [7–10, 28–31] and that of the 2015 Middle East Respiratory Syndrome (MERS) [32] and 2003 Severe Acute Respiratory Syndrome (SARS) outbreaks [33–36]. Implicit is the struggle they face with their perceived self-worth and their prevailing sense of having fallen short – as a physician, colleague, mother, daughter, person [37, 38]. This is perplexing as burnout, post-traumatic stress, depression and anxiety disorders are already commonplace in their ordinary line of work [39–43]. When unaddressed, these issues reportedly lead to higher rates of professional medical errors and malpractice, personal substance abuse, divorce, early retirement, self-harm and suicide [44–50]. Intruding on their usual thoughts and functioning, this crisis has potentially all-consuming effects on their personhood and may exacerbate these poor outcomes.

Personhood here broadly refers to one’s state of being human – an entity conferred full moral status, dignity and the right to life [51–53]. Well beyond the scope of this paper, comprehensive overviews and diverse perspectives have been offered by Carrithers et al. (1985) [54], Sarah Bishop Merrill (1998) [55] and Huyssteen et al. (2011) [56]. However only one framework to date offers rigorous accounts of personhood with a comprehensive approach towards existential, individual, relational and social concepts of the self – one that accounts for flux and change, influenced by internal and external sources of duress.

### The Ring Theory of Personhood: a relevant, responsive framework

Krishna and Alsuwaigh's (2015) Ring Theory of Personhood (RToP), originally designed for the palliative care setting, is adopted to explain COVID-19’s impact on personhood as it serves as a dynamic, clinically-evidenced and holistic framework [57–59]. Personhood is broadly defined here as ‘what makes you, you’. Although particularly pertinent to issues surrounding euthanasia, abortion and dementia care [60–65], for the sentient person, it also calls into question one’s personal belief in their right to life and willingness or ability to live. The RToP captures evolving concepts of personhood through porous circles that correspond with one’s Innate, Individual, Relational and Societal Ring. The RToP draws attention to whether the elements that presently ‘make you, you’ will suffice to bolster you in the face of extended suffering or if they serve as sources of duress themselves.

#### Innate ring

The Innate Ring comprises the inalienable aspects of human beings – their being alive, their genetic makeup endowing them with human features and their connections with the Divine. Lost only upon death, these serve as the Core of their personhood.

As death often inspires fear, particularly when one’s own is intimated, confrontation with one’s own mortality threatens the integrity of this fundamental self-concept. The human features the physicians share with the grievously ill reinforces their common fallibility, foreshadowing not only an end, but a potential trajectory that sees them wasting away. Death anxiety has high correlations to burnout, depersonalisation and absenteeism [66–69] and may lead to wavering of religious faith previously serving as a coping mechanism [70, 71].

#### Individual ring

The Individual Ring relates to the person’s conscious function. This includes their emotions, cognitive thoughts, values, beliefs, hopes and their ability for cogitation, communication and action. As such, emotional, cognitive and behavioural responses to disruptions within the other rings are often made manifest here where consciousness, self-awareness and human capacities for self-expression reside.

Emotional, psychological and physical ramifications such as personal guilt, sorrow, fatigue and feelings of helplessness and unpreparedness in the professional domain are expressed here. Closely associated are distressing thoughts, decision-making and forced adaptations, out of the physician’s locus of control. Whilst professional roles and obligations are established in the wider Societal Ring in accordance with institutional and enculturated expectations, it is difficult for one to fully separate their personal values, beliefs and hopes from their professional selves. With entwined self-concepts affected, heightened despair may result in maladaptive coping strategies such as indulging in alcohol, gambling or avoidance behaviours such as emotional withdrawal if appropriate avenues are not made known [72–75].

#### Relational ring

The Relational Ring comprises close relationships, a privilege conferred to others by virtue of their being family, or established through personal and positive interactions. This extends to good friends or even close colleagues and their importance is determined by the person themselves.

One’s concept of personhood is also keenly associated to the joys and afflictions experienced with these close relations. These enduring ties forged by mutual affection, dependence and the desire for mutual beneficence are upheld through the fulfilment of explicit or implicit duties. With the intent of minimising harm and the spread of infection, however, drastic modifications have been made to family and home living arrangements, interfering with the physician’s ability to provide comfort and support to their loved ones. Reduced accessibility to usual support systems may further intensify their grief and sense of isolation beyond the physical [76, 77].

#### Societal ring

The Societal Ring comprises of less intimate relationships such as more distant family, friends, colleagues and members of the wider community. In addition, it also contains enculturated roles and expectations that the person is bound to by virtue of their presence within society. It obliges the person to comply with legal, ethical and sociocultural standards.

This ring notably confers to physicians their basic rights and social value. It is clear that care owed to them by local governments and host institutions have been  lacking severely, with evidence rooted in poor national responses to the viral outbreak and injustice entrenched in prevailing healthcare systems. The ignorance and hostility of the wider community also threatens to undermine them as persons deserving dignity and respect. As social creatures, membership and approval deeply influence one’s self-concept and sense of worth. Societal indifference or malevolence may heighten feelings of insignificance and inadequacy [18, 78–81].

#### Adaptive and ‘compensatory’ nature of rings

The greatest strength of the RToP framework is that it encaptures personhood as an individualised, situation-specific and evolving concept. Here, sizes of the rings correspond to the number of elements contained within them, the most important positioned closest to the centre. If long-standing spiritual beliefs once ingrained no longer have significant bearings on the physician’s personhood, this element may be removed from their Innate Ring, thus shrinking its size. If colleagues once distant grow closer due to shared harrowing experiences in the COVID-19 pandemic, these colleagues may shift from the Societal Ring to the Relational Ring.

**Fig. 1:**
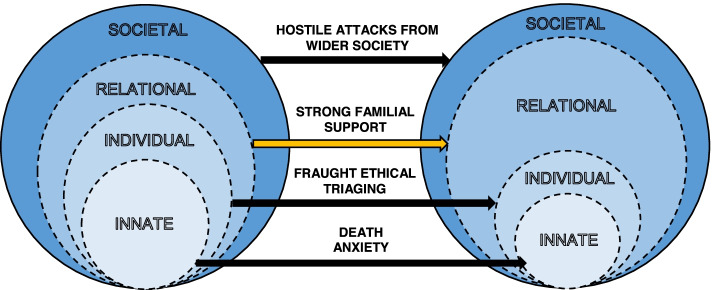
Compensatory Nature of Rings

In addition, the presence of coping strategies account for the rings’ ‘compensatory’ nature. Whilst various forms of suffering serve as tensions that threaten the integrity of specific rings, coping strategies may serve as ‘buffers’ which continue to fortify the physician’s overall concept of personhood. An example is visualised in Fig. 1 where the overall size of the rings are maintained despite internal changes. As death anxiety threatens their spiritual faith, the Innate Ring shrinks in size and influence. As fraught ethical triaging disintegrates the cogency of their moral values and as racial attacks threaten their societal membership, the Individual and Societal Ring also shrink. However, the presence of strong intercollegial support from close co-workers may imbue them with a sense of personal value through validation of their efforts. The Relational Ring thus successfully buffers against loss of integrity in the other rings. This encapsulates the paramount importance of providing physicians with timely and targeted support.

### Providing timely, targeted support

Studies have underscored the pivotal role of healthcare institutions and offered cogent means of bolstering the psychological resilience of physicians amidst this time of crisis [7–9, 30, 33]. Adopting the RToP framework and building upon salient themes drawn from these reflections, a comprehensive assessment tool could be developed, validated and used to quickly detect at-risk physicians with compromised self-concepts and poor coping strategies. For if prolonged, these may amplify their risk of psychopathological disorders and behaviours highlighted earlier. From an established list, physicians could identify prevailing elements within each of their rings, their relative importance to one another and the sources and strength of duress and buffers in place. Whilst clinical tools such as the Hospital Anxiety and Depression Scale (HADS) [82], PTSD Scale-Self Report for DSM-5 (PSS-SR5) [83] and UConn Racial/Ethnic Stress & Trauma Survey [84] are scaled based on frequency or intensity of specific psychological states or events, the RToP tool instead offers a visual manifestation of their overarching personhood by illustrating the general integrity of each ring – either significantly weakened by personal sufferings or thickened by buffers established. Here, healthcare institutions could administer this tool and direct susceptible physicians to a dedicated team of medical social workers for urgent, targeted intervention. Personal coping strategies such as positive reframing and the various practices of self-care outlined in Table 1 may be further encouraged.

As the full effects of this devastating pandemic may only be realised in years to come, the novel tool may also serve as a longitudinal intervention used to assess the presence and effectiveness of buffers established by local healthcare institutions. Many deficiencies have been highlighted in these reflections. These include the timely provision of adequate personal protective equipment, swab testing for high-risk physicians and up-to-date briefs to minimise feelings of professional impotence. In addition, childcare arrangements and lodging for those isolating from their families have proven to be much needed. As support should not only be reactive but proactively designed, nurturing intercollegial solidarity should be prioritised as it offers opportunities for role modelling and the mutual trading of life experiences, advice and lighthearted camaraderie. The institution of such support structures will affirm these physicians that their needs are valid and their lives valued.

## Conclusion

Framed through The Ring Theory of Personhood, it is clear that the COVID-19 pandemic has not only dramatically altered the day to day routines of physicians, but that these changes have had detrimental impact on intrinsic perceptions of who they are and their value to others. Whilst it is clear that social media platforms are brimming with insight and offer remarkable opportunities for self-expression, negotiation and a re-establishment of control, speaking up on such public interfaces as a healthcare professional is still contentious at best. The very act of posting on these platforms underlines a desire for discourse, interaction and validation. If local healthcare institutions offer timely, targeted support and actively avail avenues for honest conversations, this may ameliorate their strong need to express their personal grievances online. Simply put, physicians are requesting to be heard. Amidst the bleakness of their days, addressing their concerns head-on may offer them a greater semblance of hope and strengthen their self-concepts, self-esteem and self-worth.

## Supplementary Information

**Additional file 1: Appendix A.** Search Strategy. **Appendix B.** PRISMA Flowchart.

## Data Availability

All data generated or analysed during this study are included in this published article and its supplementary information files.

## Abbreviation

RToP: Ring Theory of Personhood
