# Supplementary material for: Changing self-concept in the time of COVID-19: a close look at physician reflections on social media
Source: Philos Ethics Humanit Med. 2022 Jan 26;17:1. doi: 10.1186/s13010-021-00113-x (PMC8789479; doi:10.1186/s13010-021-00113-x)

Appendix A: Search Strategy

(COVID-19 OR COVID OR coronavirus OR pandemic) AND (doctor OR doctors OR physician OR physicians) AND (reflection OR reflections OR experience OR experiences OR self OR image OR life OR lives OR affect OR effect OR effects OR impact OR impacts OR personal OR professional)

Appendix B: PRISMA Flowchart

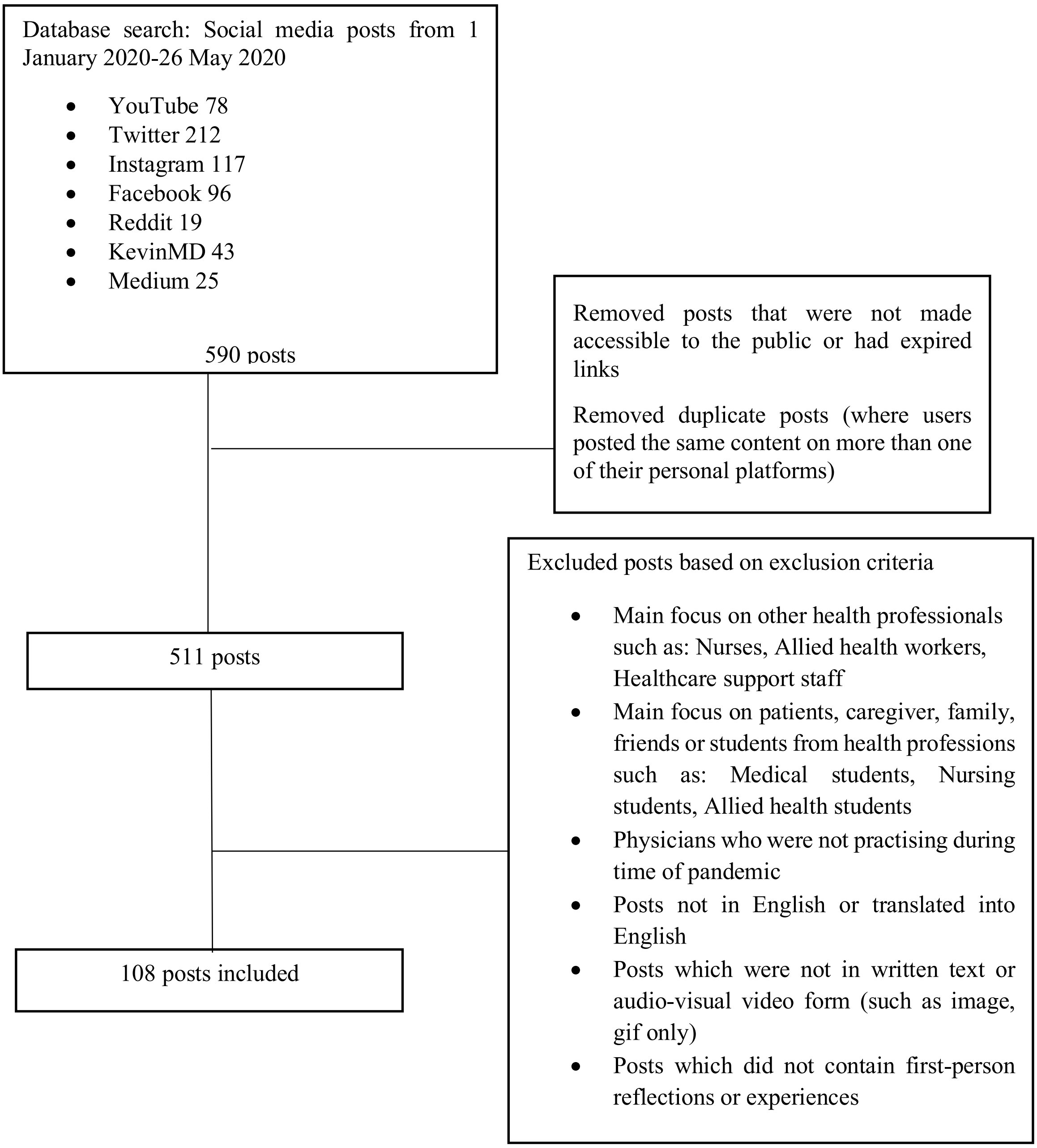

Supplement: Supplementary file 1 — Additional file 1: Appendix A. Search Strategy. Appendix B. PRISMA Flowchart. [file 13010_2021_113_MOESM1_ESM.pdf]
